# Brazilian Protocol for Sexually Transmitted Infections, 2020: infections causing vaginal discharge

**DOI:** 10.1590/0037-8682-593-2020

**Published:** 2021-05-17

**Authors:** Newton Sergio de Carvalho, José Eleutério, Ana Gabriela Travassos, Lutigardes Bastos Santana, Angélica Espinosa Miranda

**Affiliations:** 1 Universidade Federal do Paraná, Departamento de Tocoginecologia, Curitiba, PR, Brasil.; 2 Universidade Federal do Ceará, Departamento de Saúde da Mulher, da Criança e do Adolescente, Fortaleza, CE, Brasil.; 3 Universidade do Estado da Bahia, Departamento de Ciências da Vida, Salvador, BA, Brasil.; 4 Ministério da Saúde, Secretaria de Vigilância em Saúde, Brasília, DF, Brasil.; 5 Universidade Federal do Espírito Santo, Departmento de Medicina Social, Vitoria, ES, Brasil.

**Keywords:** Vaginitis, Candidiasis, Vulvovaginal, Vaginosis, Bacterial, Trichomonas Infections, Sexually transmitted diseases

## Abstract

The topic of vaginal discharge is one of the chapters of the Clinical Protocol and Therapeutic Guidelines for Comprehensive Health Care for People with Sexually Transmitted Infections, published by the Brazilian Ministry of Health in 2020. The chapter has been developed based on scientific evidence and validated in discussions with specialists. This article presents epidemiological and clinical aspects associated with vaginal discharge conditions, as well as guidance to health service managers and health professionals. Screening, diagnosing, and treating these conditions, the main complaints among women seeking health services, caused by infectious or non-infectious factors, also are presented. Besides, information is presented on surveillance, prevention, and control actions to promote knowledge of the problem and provide quality care and effective treatment.

## Highlighted excerpt

In healthcare servicing cases of sexually transmitted infections, vaginal discharge is the main referred symptom, common among pregnant women.

## INTRODUCTION

This article approaches the chapter on infections causing vaginal discharge in the Clinical Protocol and Therapeutic Guidelines for Comprehensive Health Care (PDCT) for People with Sexually Transmitted Infections (STI), published by the Health Surveillance Department of the Brazilian Ministry of Health. For elaborating the PCDT, a selection and analysis of the evidence available in the literature were performed, and a panel of specialists discussed it. The document was approved by the National Committee for Technology Incorporation to the Brazilian National Health System (Conitec) and updated by the team of specialists in STI in 2020^1^. 

## EPIDEMIOLOGICAL ASPECTS

 In clinics addressing STI cases, vaginal discharge is the main referred symptom^2^ ^-^ ^4^, being also a frequent complaint in pregnant women^5^ ^-^ ^7^. Non-infectious causes of vaginal discharge include excessive elimination of physiological mucous material, presence of intravaginal foreign objects, and atrophic vaginitis. It can occur in women after menopause, during breastfeeding, or as an effect of local radiotherapy in oncology treatment^4^ ^,^ ^7^ ^,^ ^8^. Other situations can cause vulvovaginal pruritus without discharge, such as allergic or irritant dermatitis (soap, perfume, and latex) or skin diseases (atopic dermatitis, lichen, and psoriasis)^8^.

Among the infectious causes of vaginal discharge, women can simultaneously present infection by more than one etiologic agent, which causes nonspecific aspect discharge^4^. The agents can be associated with vaginitis or vaginosis, depending on the existence or nonexistence of the inflammatory process. These are conditions of the vulvovaginal stratified epithelium. The most frequent etiologic agents are fungi (mainly *Candida albicans*), anaerobic bacteria associated with bacterial vaginosis, and protozoan *Trichomonas vaginalis*. There can also be cytolytic vaginosis, dysbiosis arising from a significant increase in lactobacilli and the lytic action on squamous cells^4^ ^,^ ^9^, and the possibility of mixed vaginitis.

**FIGURE 1:**
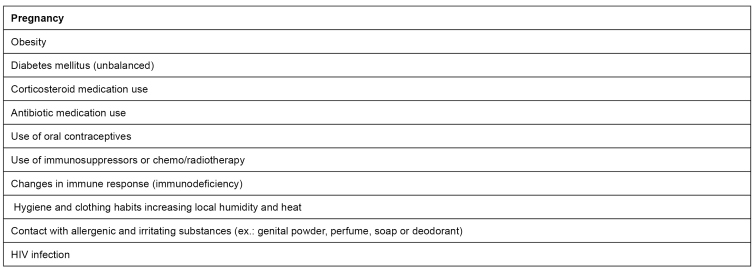
Factors predisposing vulvovaginal candidiasis.

**Source:** adapted from the Clinical Protocol and Therapeutic Guidelines for Comprehensive Health Care for People with Sexually Transmitted Infections, 2020^22^.

*C. albicans* is the etiological agent of vulvovaginal candidiasis in 80% to 92% of cases; non-*albicans* species (*Candida glabrata, Candida tropicalis, Candida krusei, Candida parapsilosis*) and *Saccharomyces cerevisae* are less prevalent^9^. Over reproductive life, 10 to 20% of women will be colonized by *Candida sp.* in an asymptomatic way, not requiring treatment, as yeast can be part of the vaginal environment^10^ ^,^ ^11^. Among factors predisposing vulvovaginal candidiasis, the ones indicated in Figure 1 are highlighted. Vulvovaginal candidiasis is classified as non-complicated and complicated. The first one occurs when all following criteria are present: mild/moderate symptoms and rare frequency; *C. albicans* as an etiologic agent; and lack of comorbidities. Complicated vulvovaginal candidiasis takes place when at least one of the following criteria are present: intense symptoms; recurrence of four or more episodes in a year; non-*albicans* etiologic agent (*C. glabrata, C. kruzei*); presence of comorbidities such as diabetes and human immunodeficiency virus, HIV; or pregnancy^4^ ^,^ ^11^. Most vulvovaginal candidiasis is not complicated and respond to different treatments. Nevertheless, we observe the recurrent form of this disease in 5% of women^11^ ^,^ ^12^.

Bacterial vaginosis is the most frequent abnormality in the lower genital tract among women in reproductive age. It is the most prevalent cause of vaginal discharge with fetid odor. It is associated with the reduction of lactobacillus and the growth of several anaerobic and facultative bacteria, such as Gram-variable short bacilli, short Gram-negative bacilli, and anaerobic Gram-negative cocci, with variation, mainly *Gardnerella, Atopobium, Prevotella, Megasphaera, Leptotrichia, Sneathia, Bifidobacterium, Dialister, Mobiluncus, Ureaplasma, Mycoplasma* and three species of *Clostridium* known as bacterial vaginosis associated bacteria, BVAB 1 to 3^13^. Certain changes in vaginal microbiome (dysbiosis) can be associated to higher frequency of bacterial vaginosis. A study on the characteristics of Brazilian women’s microbiome in reproductive age showed the microbiome type (community-state types, CST) corresponding to CST IV, with depletion of lactobacillus and growth in vaginal pH in 27.4%, with bacterial vaginosis present in 79.6% of the cases^14^.

Trichomonas infections are the most common non-viral STI, being present in 140 million people in the world. A flagellated parasite causes it, *T. vaginalis* ^15^, that causes changes to the vaginal microbiome, growth of local inflammatory response, and relevant reduction in the number of *Lactobacillus sp.* Trichomonas infections are associated with an increasing of the probability of HIV transmission^16^.

In some cases, there is a growth of lactobacillus, with great destruction of intermediate squamous cells (cytolysis), associated with genital irritative symptoms^17^. It is cytolytic vaginosis, a situation that is usually cyclic in women in reproductive years^16^, with a prevalence of 1% to 7%, most frequent for 25 and 40 years^17^ ^,^ ^18^.

Mixed vaginitis is a condition in which two pathogens are causing vulvovaginal symptoms. They can be pathogens with vaginal pH preferences equal to or not to one another. There can be, for example, vaginitis caused by *T. vaginalis* associated with bacterial vaginosis^19^. Notwithstanding, the most frequent form of mixed vaginosis is the association of *Candida* infection with bacterial vaginosis. Its frequency varies between 7% and 22% of vaginal discharge cases, depending on the diagnosis method used^20^.

## CLINICAL ASPECTS

Vaginal infection and dysbiosis can be associated with different discharge forms, pruritus, irritation, and pain^21^. Hence, it is essential always to identify, in anamnesis, aspects related to consistency, color, and modifications in vaginal discharge, in addition to the presence of pruritus, local irritation and smell. The investigation of clinical history must be detailed, encompassing information on sexual behavior and practices, date of last menstrual period, vaginal hygiene practices, use of topical or systemic medication and other possible local irritating agents, in addition to comorbidities such as diabetes and HIV infection^22^. During the gynecologic examination, the healthcare professional must identify characteristics of vaginal flow as observed in speculum examination and alterations present, such as inflammation (colpitis), ulcer, edema, and erythema^22^.

### Vulvovaginal candidiasis

Characteristic signs of vulvovaginal candidiasis are erythema, vulvar fissures, clumpy discharge, white plates stuck to the vaginal wall, vulvar edema, excoriations, etc., satellite lesions, which can become pustules through intense scratching^8^. Usually, there is an association between vaginitis and vulvitis, although such conditions can also take place separately. Clinically, vulvovaginal candidiasis can associate with vaginal introitus dyspareunia and external dysuria due to irritation and local lesions^21^.

### Bacterial vaginosis

On the other hand, in bacterial vaginosis, women present homogeneous and fluid vaginal discharge, frequently with fetid odor. Unbalances in the vaginal microbiome have been identified as an alteration often associated with some STIs, including HIV, complications in gynecological surgeries, and pregnancy (premature rupture of membrane, chorioamnionitis, prematurity, and post-cesarean endometritis). When it is present during invasive procedures, such as uterine curettage, endometrial biopsy, and insertion of an intrauterine device (IUD), bacterial vaginosis can increase pelvic inflammatory disease risk^13^. The condition has also been associated with higher risk of human papillomavirus (HPV), and precancerous cervical lesions^23^. The reduction of commensal lactobacillus is associated with increased vaginal pH and the growth of anaerobic microbiota, with amino production (putrescine, cadaverine, and trimethylamine) that become volatile when mixed with substances of alkaline pH, releasing these enzymes to the environment and producing an unpleasant odor. This situation happens particularly after intercourse and menstruation (which alkalinizes vaginal contents), contributing to women’s main complaint. In speculum examination, we can observe that the vaginal walls are brown, most integral, and homogeneous to Schiller’s test, bathed in pearly bubbly discharge^24^.

### Trichomonas infections

Signals and symptoms of trichomonas infections are comprised of greenish-yellow, sometimes grayish, bubbly, and foamy intense vaginal discharge, with fetid odor and possible pruritus. It can occur bleeding in sexual intercourse and dyspareunia associated with the inflammatory process in more serious cases. It can also occur vulvar edema and urinary symptoms, such as dysuria^25^. Most of the cases of trichomonas infections are asymptomatic and stay without diagnosis or treatment^26^. Although the process is not entirely comprehended, and the protozoan can make easier the transmission of other more aggressive infectious agents, facilitate the evolution to pelvic inflammatory disease and bacterial vaginosis. In pregnancy, when it is not treated, it can be associated with premature rupture of the membranes^27^. In speculum examination, microulcerations are commonly seen in the cervix, similarly to the aspect of a strawberry or raspberry (Schiller’s test with “tiger” or “leopard” aspect). Trichomonas infections can be associated with bacterial vaginosis in an anaerobic environment, with amines being volatilized with its suggestive odor^15^.

### Cytolytic vaginosis

In cases of cytolytic vaginosis, at first, the symptoms are very similar to the ones for vulvovaginal candidiasis, when the woman refers to genital irritation associated with a white to yellowish-white vaginal discharge with lumps, but frequently with cyclic behavior^9^.Speculum examination shows white, milky, and lumpy vaginal content stuck to the vaginal walls. Vaginal pH is presented as lower than 4.5, and the test for amines shows negative^18^. On the other hand, in mixed vaginitis, the clinical picture varies, depending on the agents encompassed. In bacterial vaginosis and candidiasis, both odorous discharge and genital pruritus can be the chief complaint^19^.

## DIAGNOSIS

**FIGURE 2:**
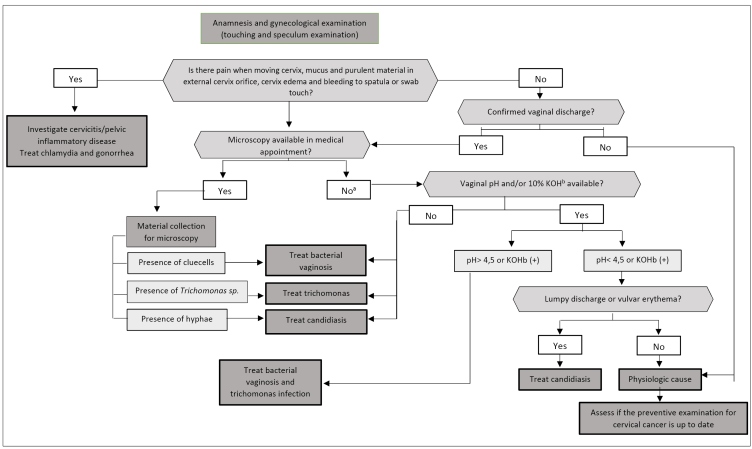
Recommendations for handling vaginal discharge.

**Source:** adapted from the Clinical Protocol and Therapeutic Guidelines for Comprehensive Health Care for People with Sexually Transmitted Infections, 2020^22^.

**Note:** a) When the collection is conducted for microscopy through Gram-staining, consider the result for managing the case; b) potassium hydroxide.

Clinical management of women with vaginal discharge is presented in Figure 2. For diagnosis clarification of the agents causing vaginal flow, we need anamnesis and supplementary examinations^19^.

### Vulvovaginal candidiasis

Vulvovaginal candidiasis diagnosis must be confirmed through laboratory examinations. The simplest one is the microscopic examination of fresh vaginal content. In this procedure, a sample of the material collected from the vaginal wall is placed on a plate. One to two drops of saline or 10% potassium hydroxide for better evidencing morphotypes of yeasts are added^28^. In addition to this examination, another simple method and low-cost is the Gram-stained vaginal smear bacterioscopy^29^. In cases of recurrent candidiasis, a culture for fungi can be needed (Sabouraud, Nickerson’s, or Microstix-Candida media) in a vaginal sample, aiming at identifying the species of fungi^30^. For a differential diagnosis of recurrent vulvovaginal candidiasis, we must consider lichen sclerosus, vulvar vestibulitis, vulvar dermatitis, vulvodynia, cytolytic vaginitis, desquamative inflammatory vaginitis, atypical forms of genital herpes, and hypersensitivity reactions^11^. 

### Bacterial vaginosis

**FIGURE 3:**
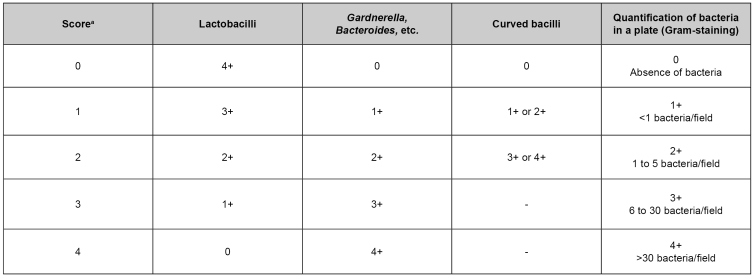
Nugent score for bacterial vaginosis diagnosis.

**Source:** Clinical Protocol and Therapeutic Guidelines for Comprehensive Health Care for People with Sexually Transmitted Infections 2020^22^.

**Note:** Result interpretation: 0 to 3 - negative for bacterial vaginosis; 4 to 6 - changed microbiota; 7 or more - bacterial vaginosis.

Bacterial vaginosis diagnosis is based on Amsel’s criteria^31^, with the need for the diagnosis, complying with three of the four following criteria: pH higher than 4,5; grayish and homogeneous vaginal discharge; positive amine discharge; and identification of clue cells in microscopic examination. The Nugent score has replaced this diagnosis criterion^13^ ^,^ ^32^. Both criteria can be associated, although the gold pattern is the Nugent laboratory procedure, using Gram staining and objective score system, indicated as evidence II-2^32^ ^,^ ^33^. This evaluation attributes a score for three morphotypes: lactobacilli, Gram-variable coccobacilli, and Gram-negative curved bacilli. After the sum of all agents’ scores, 7-10 indicates bacterial vaginosis, 4-6 is intermediate, and 0-3 is normal^32^ (Figure 3). 

### Trichomonas infections

In trichomonas infections, the most used laboratory microbiological diagnosis in clinical practice is the microscopic examination of fresh vaginal content in saline, observing the parasite in the microscope. The protozoan movement, a flagellated one, can be seen, as well as a significant number of leukocytes. pH is almost always higher than 5.0^29^. In most cases, the test for amines is positive, and we can observe Gram-negative bacteria in bacterioscopy when there are tests for diagnosis with Gram-staining. On the other hand, *T. vaginalis* is a flagellated protozoan stained through Papanicolaou or Giemsa techniques. Culture can be requested in cases of challenging diagnosis. Culture media vary, and they can include Diamond’s, Trichosel, and InPouch TV^34^. The diagnosis can also be conducted with molecular biology through a polymerase chain reaction, PCR, including multiplex tests that can detect more than one pathogen and allow for identifying even asymptomatic cases^15^, due to its high sensitivity.

The current pattern of diagnosis test for vulvovaginitis depends on the structure available at the place of attendance. Most of the diagnoses are conducted empirically and based on clinics, although the availability of a microscope for fresh examination is an important supplementary examination. Molecular tests directed to bacterial vaginosis diagnosis, *Candida sp.* and *T. vaginalis* can improve diagnostic accuracy and reduce result time compared to culture^35^ ^,^ ^36^. This can be especially important for bacterial vaginosis, which encompasses multiple organisms in vaginal microbiota^37^.

### Cytolytic vaginosis

Cytolytic vaginosis diagnosis must comply with the following criteria: white discharge, pruritus or genital burning, vaginal pH between 3.5 and 4.5, and examination of fresh vaginal content without any pathogen, with identification of a significant population of medium bacilli, some naked nuclei, and cell detritus^38^. Gram bacterioscopy and Papanicolaou examination can present the same microscopic findings^22^.

In cases of mixed vaginitis, the presence of two microorganisms in the same moment not necessarily implies that both are pathogenic, especially when it comes to bacterial vaginosis and vulvovaginal candidiasis, considering that both yeast and bacteria in bacterial vaginosis not always cause the disease. Therefore, it is important to differentiate between mixed vaginitis and co-occurrence^18^. In the first case, both the agents are pathogenic, which does not necessarily occur in the second case, as the change in the vaginal microbiome can be the agent causing a recurrence. More advanced methods, such as PCR, can lead to inconclusive results if they are not correctly interpreted. Cases with bacterial vaginosis criteria presenting identified inflammatory infiltration were sometimes identified as suggesting a picture of mixed vaginitis^39^. The association can be observed using fresh examination, bacterioscopy, cytology, or molecular biology^40^.

### Treatment

**FIGURE 4:**
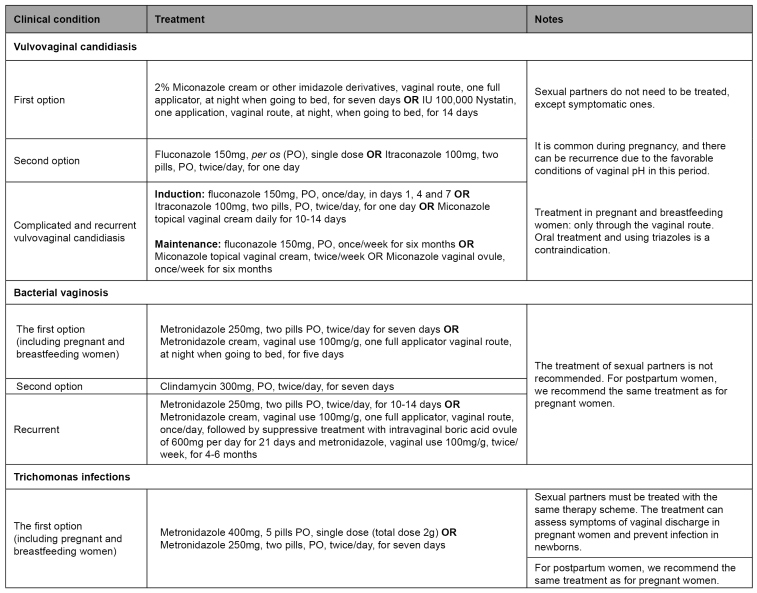
Treatment of vulvovaginal candidiasis, bacterial vaginosis, and trichomonas infections.

Source: adapted from the Clinical Protocol and Therapeutic Guidelines for Comprehensive Health Care for People with Sexually Transmitted Infections, 2020^22^.

Options for treating the vulvovaginitis caused by *Candida*, bacterial vaginosis, and trichomonas infections are described in Figure 4. It is crucial to suspend sexual relations to prevent contaminations during treatment, which must be kept during the menstrual period. In the treatment with metronidazole, drinking alcoholic beverages must be avoided, due to the antabuse effect, caused by the interaction with imidazole derivatives with alcohol and characterized by discomfort, nausea, vertigo, and a metallic taste in the mouth^22^.

Cytolytic vaginosis is treated through using sodium bicarbonate^38^ in vaginal baths (15g to 30g of sodium bicarbonate in 0.5L of warm water), twice to three times a week, for two to six weeks^18^. The treatment on cases of mixed vaginitis uses concomitant therapy for each of the pathogens. In case of candidiasis and vaginosis, using antifungal medications with metronidazole is recommeded^19^.

It is essential to highlight that trichomonas infections can change the evaluation of oncological cytology. In cases in which cellular morphological alterations and trichomonas infections, the treatment must be conducted, and the cytology repeated after three months to identify if the changes persist^22^. In recurrent bacterial vaginosis, the triple regime (use of metronidazole cream for ten days, followed by boric acid for 21 days and maintenance with metronidazole cream twice a week, for four to six months) seems successful albeit requiring validation by randomized and controlled clinical assays. The role of boric acid is to remove the bacterial biofilm formed on the vaginal wall that facilitates the persistence of such a picture^41^.

In the vulvovaginal candidiasis cases that are recurrent or difficult to control, we must investigate predisposing systemic causes, such as diabetes, immunodepression (including HIV infection), and the use of corticosteroids. Among the rare adverse reasons (0.01% to 0.1%) of fluconazole use, we can cite agranulocytosis, leukopenia, neutropenia, thrombocytopenia, anaphylaxis, angioedema, hypertriglyceridemia, hypercholesterolemia, hypokalemia, toxicity, and hepatic insufficiency; for this reason, it is essential to investigate liver functioning^29^.

There is no recommendation for screening bacterial vaginosis in asymptomatic women. The treatment is recommended for all symptomatic women with the potential risk of complications, as before IUD insertion, gynecological surgeries, and invasive examinations of the genital tract. The treatment must be simultaneous to the procedure, with no reason for suspension or delay^22^. Recurrence of bacterial vaginosis after treatment is common: around 15% to 30% of women present symptoms for the period of 30 to 90 days after antibiotics therapy, while 70% of women experience a recurrence within nine months^42^ ^,^ ^43^. Some factors justify the lack of therapeutic response to the conventional schemes; among them, frequent sexual activity with no use of condoms, vaginal douche, UID use, improper immune response, and bacterial resistance to imidazole medication. Strains of *Atopobium vaginae* resistant to metronidazole medication are identified in different women with recurrent bacterial vaginosis; however, such bacilli are sensible to clindamycin and cephalosporins^22^ ^,^ ^41^.

The recurrence of infection by *T. vaginalis*, whose cause is still not clear, occurs between 5% and 31% of treated women. We need to assess the partner’s treatment, the exposure to new partners, and, finally, the therapeutic failure^44^. Treatment with a single dose and the presence of HIV infection seem to be the factors most associated with therapeutic failure^45^. The molecular mechanism of clinical resistance by *T. vaginalis* is not clear^44^.

In the follow-up of women with *Candida* infections, we can observe an increased frequency of recurrences in the presence of changes in cell immunity, such as in women living with HIV or diabetes and those with *Candida* non-*albicans* infections. In combination with behavioral changes, prolonged treatment has been used with some response in the treatment of recurrences^46^.The etiological diagnosis is vital in recurrence cases, aiming at identifying the species present and confirm fungal infection, considering the existence of differential diagnoses such as cytolytic vulvovaginitis, allergic reactions, and mixed infections^47^.

## SURVEILLANCE, PREVENTION, AND CONTROL

STI diagnosis, different from endogenous and iatrogenic infections, implies the need for guidance and treatment of sexual partners. It is essential to assess the woman’s perception of the existence of physiological vaginal discharge and recommend investigating other STI^22^.

The treatment of sexual partners, when recommended, must be conducted ideally face-to-face, with the due guidance, request of examinations for diagnosis of other STI and identification, summoning and treatment of other different sexual partners, aiming at blocking the chain of infection^22^. In trichomonas infections, as it is an STI, we recommend treating the sexual partners with the same therapeutic scheme as the diagnosed case^47^. In cases of bacterial vaginosis, the sexual partners do not need to be treated. In situations of vulvovaginal candidiasis, the partners only need to be treated if they present symptoms. However, we need to emphasize the role of counseling to the sexual partners^22^.

Cases of infections causing vaginal discharge do not present compulsory notification at the national level, and trichomonas infections can be included, if it is considered convenient, in the list of reports of municipalities and states.

## SPECIAL POPULATIONS

### Pregnant women

Vulvovaginal candidiasis is common during pregnancy, and there can be recurrence due to the favorable conditions of vaginal pH in this period. The treatment in pregnant and breastfeeding women must be conducted only through the vaginal route. Oral treatment and the use of triazole medications is contraindication^22^ ^,^ ^48^. Although a systematic review showed a lack of benefits in tracing bacterial vaginosis in asymptomatic pregnant women^49^, other studies showed advantages when the disease is associated with other agents^50^. Despite being controversial, there are suggestions for the treatment of asymptomatic pregnant women. The benefit is defined for those with preterm delivery history with comorbidities. 

### HIV infections

The treatment must be conducted with the usual schemes for vulvovaginal candidiasis, bacterial vaginosis, and trichomonas infections^22^, but drug interaction between metronidazole and ritonavir must be observed which can increase nausea and vomit occurrence, reducing antiretroviral adherence. To avoid this occurrence, we recommend a two-hour interval between the administrations of both medications. Bacterial vaginosis has also been observed to provide a set of microorganisms that can increase the levels of viral copies of genital HIV and make vulvovaginal candidiasis episodes more severe and complicated^51^.
